# Apigenin and luteolin modulate microglial activation via inhibition of STAT1-induced CD40 expression

**DOI:** 10.1186/1742-2094-5-41

**Published:** 2008-09-25

**Authors:** Kavon Rezai-Zadeh, Jared Ehrhart, Yun Bai, Paul R Sanberg, Paula Bickford, Jun Tan, R Douglas Shytle

**Affiliations:** 1 Silver Child Development Center, Department of Psychiatry and Behavioral Medicine, College of Medicine, University of South Florida, Tampa, FL 33612, USA; 2 Center Excellence in Aging and Brain Repair, Department of Neurosurgery, College of Medicine, University of South Florida, Tampa, FL 33612, USA; 3 James A. Haley Veterans' Hospital, Tampa, Fl 33612, USA

## Abstract

**Background:**

It is well known that most neurodegenerative diseases are associated with microglia-mediated inflammation. Our previous research demonstrates that the CD40 signaling is critically involved in microglia-related immune responses in the brain. For example, it is well known that the activation of the signal transducer and activator of transcription (STAT) signaling pathway plays a central role in interferon-gamma (IFN-γ)-induced microglial CD40 expression. We and others have previously reported that microglial CD40 expression is significantly induced by IFN-γ and amyloid-β (Aβ) peptide. Recent studies have shown that certain flavonoids possess anti-inflammatory and neuroprotective properties distinct from their well-known anti-oxidant effects. In particular, flavonoids, apigenin and luteolin have been found to be effective CD40 immunomodulators.

**Methods:**

Cultured microglia, both N9 and primary derived lines, were treated with flavonoids in the presence of IFN-γ and/or CD40 ligation to assess any anti-inflammatory effects and/or mechanisms. CD40 expression on microglia was analyzed by fluorescence activated cell sorting (FACS). Anti-inflammatory effects and mechanisms were confirmed by ELISA for interlekin-6 (IL-6) and TNF-α, lactate dehydrogenase (LDH) assay, and STAT1 Western blotting.

**Results:**

Apigenin and luteolin concentration-dependently suppressed IFN-γ-induced CD40 expression. Apigenin and luteolin also suppressed microglial TNF-α and IL-6 production stimulated by IFN-gamma challenge in the presence of CD40 ligation. In addition, apigenin and luteolin markedly inhibited IFN-γ-induced phosphorylation of STAT1 with little impact on cell survival.

**Conclusion:**

Our findings provide further support for apigenin and luteolin's anti-inflammatory effects and suggest that these flavonoids may have neuroprotective/disease-modifying properties in various neurodegenerative disorders, including Alzheimer's disease (AD).

## Background

Multiple lines of evidence suggest microglia, the resident immune cells of the central nervous system (CNS), play a critical role in the etiology of various neurodegenerative diseases. Chronic activation of microglia is believed to trigger and maintain an inflammatory response, which may ultimately lead to neuronal cell death such as that observed in Alzheimer's disease (AD), HIV-dementia, Parkinson's disease, prion disease, amyotrophic lateral sclerosis, and multiple sclerosis [1-11]. In fact, this chronic activation exposes the CNS to elevated levels of a wide array of potentially neurotoxic molecules including pro-inflammatory cytokines, complement proteins, proteinases, and reactive oxygen species (ROS) [12-17]. Conversely, an alternative view suggests that dysregulation of microglial activation may prevent appropriate immune responses necessary to respond to neuroinsults [18]. Essential to microglial activation is the stimulatory signal from CD40 ligation. CD40 and its ligand (CD40L) are key immunoregulatory molecules that provide co-stimulatory input to cells from both the innate and adaptive arms of the immune system [19-22]. The classical stimulatory signal for microglial activation is propagated by T-cell release of interferon-gamma (IFN-γ), which consequently sensitizes the microglia by upregulating the expression of various immunoregulatory molecules, including CD40, on their cell surfaces [23,24]. Furthermore, it is well known that the activation of the Janus kinase/signal transducer and activator of transcription (JAK/STAT) signaling pathway plays a central role in this IFN-γ-induced microglial CD40 expression [25,26]. We have previously reported that microglial CD40 expression is significantly increased by IFN-γ in the presence of β-amyloid (Aβ) peptide via STAT1 activation [27,28]. Accordingly, modulation of the JAK/STAT signaling pathway may not only prove to be an effective means for suppressing microglial-mediated inflammation but also an important target for neurodegenerative disease therapy.

While several anti-inflammatory drugs have been found to prevent microglial-mediated inflammation, their underlying mechanisms remain unclear and the search for more effective practical compounds continues. Recent research has focused on the analysis of flavonoids, which epidemiological study suggest are beneficial against the neurodegeneration and aging processes [29-33]. Flavonoids, a group of phenolic phytochemicals, are common in vascular plants and are abundant in particular spices, vegetables, and fruits. They are considered important constituents in the human diet, although their daily intake varies with dietary habits [34,35]. Several medicinal properties have been ascribed to flavonoids, notably anti-oxidant [36,37], anti-carcinogenic [38,39], and anti-inflammatory activity [40-42]. One such flavonoid, apigenin, and its phase I metabolite, luteolin, have been found to reduce CD40 and CD40L expression on dendritic cells and basophils, respectively [43,44]. Previous research has also shown apigenin's ability to inhibit pro-inflammatory cytokines production by monocytes, macrophages, and microglia and further substantiates this compound as versatile immunomodulator [45-49].

In the present study, we investigate the potential anti-inflammatory effects and mechanisms of these flavonoids, apigenin and luteolin, in cultured microglia. Our findings demonstrate that treatment of both N9 and murine-derived primary microglia cell lines with apigenin and luteolin significantly reduces CD40 expression induced by IFN-γ. This reduction is paralleled by significant decreases in the release of the pro-inflammatory cytokines interleukin-6 (IL-6) and tumor necrosis factor-α (TNF-α) by the microglia. Furthermore, data show that apigenin and luteolin treatments achieve these reductions through inactivation of STAT1 and suggest a mechanism whereby these compounds may prove to be an effective therapy for neurodegeneration.

## Methods

### Animals and microglial cell cultures

Breeding pairs of BALB/c mice were purchased from Jackson Laboratory (Bar Harbor, ME) and housed in the animal facility at the University of South Florida, College of Medicine. Murine primary culture microglia were isolated from mouse cerebral cortices and grown in RPMI 1640 medium supplemented with 5% FCS, 2 mM glutamine, 100 U/ml penicillin, 0.1 μg/ml streptomycin, and 0.05 mM 2-mercaptoethanol according to previously described methods [50]. Briefly, cerebral cortices from newborn mice (1–2 day-old) were isolated under sterile conditions and were kept at 4°C before mechanical dissociation. Cells were plated in 75 cm^2 ^flasks and complete medium was added. Primary cultures were kept for 14 days so that only glial cells remained and microglial cells were isolated by shaking flasks at 200 rpm in a Lab-Line incubator-shaker. More than 98% of these glial cells stained positive for microglial marker Mac-1 (CD11b/CD18; Boehringer Mannheim, Indianapolis, IN; data not shown). All animal protocols were approved by the Committee of Animal Research at the University of South Florida, in accordance with the National Institutes of Health guidelines. N9 microglial originally generated from myc-immortalized mice were cultured as previously described [50,51]. Briefly, N9 microglial cells were maintained and plated in 75 cm^2 ^flasks with MEM medium supplemented with 5% FCS and 2 mM glutamine.

### Flow cytometric analysis of microglial CD40 expression

Primary cultured microglial cells were plated in 6-well tissue culture plates at 5 × 10^5 ^cells/well and incubated with apigenin and luteolin at different doses in the presence or absence of IFN-γ (100 U/ml). Eight hours after incubation, these microglial cells were washed with flow buffer [PBS containing 0.1% (w/v) sodium azide and 2% (v/v) FCS] and re-suspended in 250 μl of ice-cold flow buffer for fluorescence activated cell sorting (FACS) analysis, according to methods described previously [50]. Briefly, cells were pre-incubated with anti-mouse CD16/CD32 monoclonal antibody (clone 2.4G2, PharMingen, Los Angeles, CA) for 10 min at 4°C to block non-specific binding to Fc receptors. Cells were then spun down at 5,000 g washed 3 times with flow buffer and then incubated with hamster anti-mouse CD40-FITC or isotype control antibody-FITC (1:100 dilution; PharMingen) in flow buffer. After 30 min incubation at room temperature, cells were washed twice with flow buffer, re-suspended in 250 μL of flow buffer and analyzed by a FACScan™ instrument (Becton Dickinson, Franklin Lanes, NJ). A minimum of 10,000 cells were accepted for FACS analysis. Cells were gated based on morphological characteristics such that apoptotic and necrotic cells were not accepted for FACS analysis using CellQuest™ software (Beckton Dickinson). Percentages of positive cells (i.e. CD40-expressing) were calculated as follows: for each treatment, the mean fluorescence value for the isotype-matched control antibody was subtracted from the mean fluorescence value for the CD40-specific antibody.

### TNF-α, IL-6, and LDH analyses

Murine primary cultured microglial cells were plated in 24-well tissue-culture plates (Costar, Cambridge, MA) at 1 × 10^5 ^cells per well and stimulated for 12 hr with either IFN-γ (100 U/ml)/CD40 agonistic antibody (2 μg/ml) (R&D Systems, Minneapolis, MN) in the presence or absence of apigenin and luteolin at a range of concentrations. Cell-free supernatants were collected and stored at -70°C until analysis. TNF-α and IL-6 levels in the supernatants were examined using ELISA kits (R&D Systems) in strict accordance with the manufacturers' protocols. Cell lysates were also prepared and the BCA protein assay (Pierce Biotechnology, Rockford, IL) was performed to measure total cellular protein. Results are shown as mean pg of TNF-α or IL-6 per mg of total cellular protein (+/- SD). Cell death was measured from lactate dehydrogenase (LDH) release in the cell-free supernatants using a CytoTox 96^® ^Non-Radioactive Cytotoxicity Assay kit (Promega, Madison, WI).

### STAT1 signaling pathway analysis

Primary culture microglial cells were plated in 6-well tissue culture plates at a density of 5 × 10^5 ^cells per well and co-incubated with IFN-γ (100 U/mL) in the presence or absence of a dose range of apigenin or luteolin for 30 min. At the end of the treatment period, microglial cells were washed in ice-cold PBS three times and lysed in ice-cold lysis buffer. After incubation for 30 min on ice, samples were centrifuged at high speed for 15 min, and supernatants were collected. Total protein content was estimated using the BCA protein assay (Pierce Biotechnology). Aliquots corresponding to 100 μg of total protein was electrophoretically separated using 10% Tris gels. Electrophoresed proteins were then transferred to PVDF membranes (Bio-Rad, Richmond, CA), washed in dH_2_O, and blocked for 1 hour at ambient temperature in TBS containing 5% (w/v) non-fat dry milk. After blocking, membranes were hybridized for 1 h at ambient temperature with various primary antibodies. Membranes were then washed 3 × for 5 min each in dH_2_O and incubated for 1 hour at ambient temperature with the appropriate HRP-conjugated secondary antibody (1:1,000, Pierce Biotechnology). All antibodies were diluted in TBS containing 5% (w/v) of non-fat dry milk. Blots were developed using the luminol reagent (Pierce Biotechnology). Densitometric analysis was conducted using a FluorS Multiimager with Quantity One™ software (BioRad, Hercules, CA). For STAT1 phosphorylation, membranes were probed with a phospho-Ser727 STAT1 antibody or a phospho-Tyr701 STAT1 antibody (Cell Signaling Technology, Danvers, MA) and stripped with stripping solution and then re-probed with antibody that recognize total STAT1 (Cell Signaling Technology).

### Statistical analysis

All data were normally distributed; therefore, in instances of single mean comparisons, Levene's test for equality of variances followed by *t*-test for independent samples was used to assess significance. In instances of multiple mean comparisons, analysis of variance (ANOVA) was used, followed by *post-hoc *comparison using Bonferonni's method. Alpha levels were set at 0.05 for all analyses. The statistical package for the social sciences release 10.0.5 (SPSS Inc., Chicago, IL, USA) was used for all data analysis.

## Results

### Apigenin and luteolin suppress IFN-γ-induced CD40 expression in N9 and murine-derived primary microglial cells

**Figure 1:**
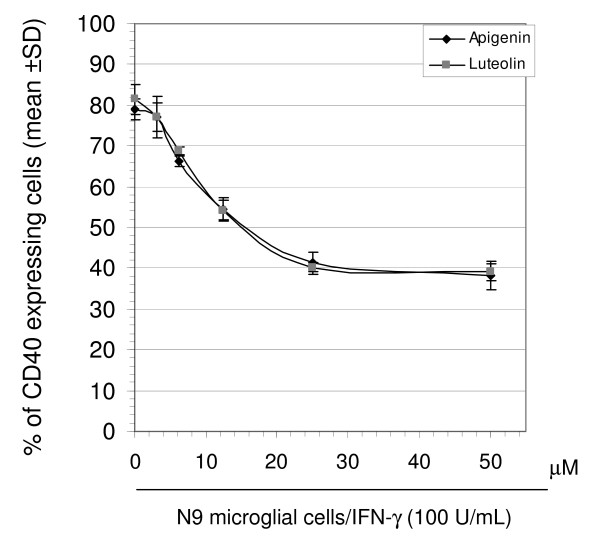
**Apigenin and luteolin reduce microglial CD40 expression induced by IFN-γ in a concentration dependent manner**. FACS analysis showed significant concentration dependent decreases in IFN-γ-induced CD40 expression by both apigenin and luteolin following 8 hrs. of co-treatment in N9 cells seeded in 6-well tissue-culture plates (5 × 10^5^/well). Data were represented as mean % of CD40 expressing cells (+/- SD). Results are representative of three independent experiments.

**Figure 2:**
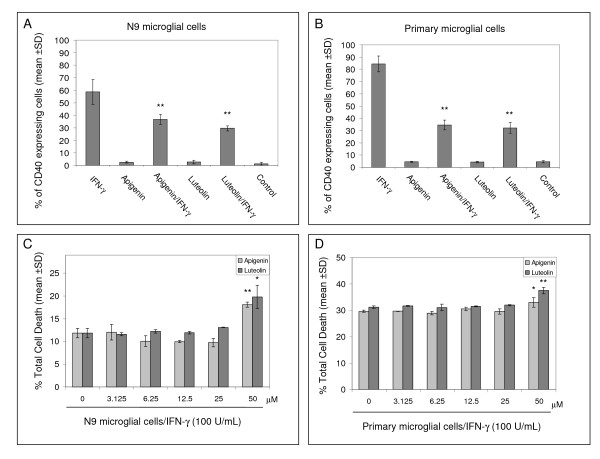
**Apigenin and luteolin inhibit microglial CD40 expression induced by IFN-γ**. N9 and murine-derived primary microglial cells were seeded in 6-well tissue-culture plates (5 × 10^5^/well) for FACS analysis and 24-well tissue-culture plates (1 × 10^5^/well) for LDH analysis in parallel. Cultured cells were co-treated with IFN-γ (100 U/mL) in the presence or absence of apigenin and luteolin (25 μM) or treated with vehicle (1% DMSO; control) for 8 hrs. For A and B, FACS analysis showed significant decreases by both apigenin and luteolin (25 μM) in IFN-γ-induced CD40 expression in N9 cells and primary microglia. Data are represented as mean % of CD40 expressing cells (+/- SD). For C and D, cultured supernatants were collected and subjected to LDH assay as indicated. Data showed no significant increase in cell toxicity below a 50 μM concentration of either flavonoid in both N9 or primary microglial cells. Data were represented as mean % of total cell death as determined by LDH present during complete lysis (+/- SD). For A, B, C and D. Results are representative of three independent experiments. (*p < 0.05; **p < 0.01).

To examine the effect apigenin and luteolin had on microglial activation via CD40, we initially treated N9 microglia cells over a range of concentrations. Following FACS analysis, we found that both compounds significantly reduced CD40 expression on the microglial cell surface (p < 0.01, Fig. 1). As illustrated in Figure 1, we clearly observe a concentration-dependent decrease in CD40 expressing cells starting at 3.125 μM through 50 μM. As these compounds have been reported to be potentially cytotoxic, we also examined the level of microglial cell death in response to apigenin and luteolin treatment by LDH assay [52]. We discovered that apigenin and luteolin treatment only induced significant cell death at a 50 μM concentration in either N9 or primary microglial cells, which along with the data from Figure 1 suggested that 25 μM may be the most efficacious concentration for regulating CD40 expression (Fig. 2C and 2D). Further, to determine if this reduction in CD40 expression continues through microglial activation, we treated both N9 and primary microglia cells with 25 μM of apigenin or luteolin in the presence of a stimulatory amount of IFN-γ as shown in figure 2. Following FACS analysis, we once more found that both compounds significantly reduced CD40 expressing cells as compared to positive control in both cell lines (p < 0.01, Fig. 2A and 2B). Taken together, the above data suggests that apigenin and luteolin may be able to modulate microglial activation via CD40.

### Apigenin and luteolin oppose the effects of IFN-γ/CD40 ligation on TNF-α and IL-6 production in N9 and murine-derived primary microglial cells

**Figure 3:**
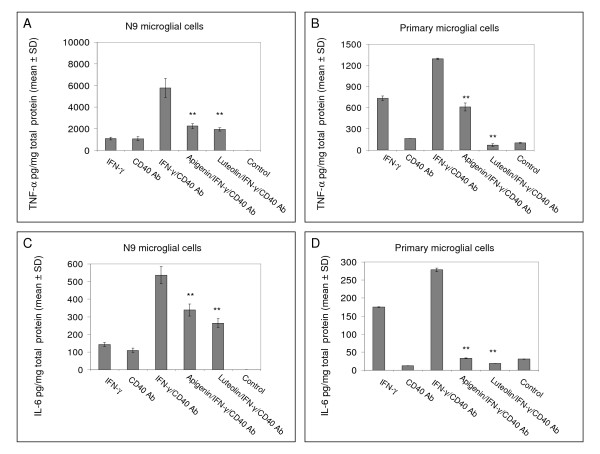
**Apigenin and luteolin oppose the effects of IFN-γ/CD40 ligation on TNF-α and IL-6 production in microglial cells**. N9 and murine-derived primary microglial cells were seeded in 24-well tissue-culture plates (1 × 10^5^/well) and co-treated with IFN-γ (100 U/mL)/agonistic anti-CD40 antibody (CD40 Ab; 2 μg/mL) in the presence or absence of apigenin and luteolin (25 μM) or treated with vehicle (1% DMSO; control) for 12 hrs. Cell cultured supernatants were collected and subjected to cytokine ELISA as indicated. Data were represented as mean pg of each cytokine in mg of total cellular protein (+/- SD). Results are representative of three independent experiments. (**p < 0.01).

To confirm the functional inhibition of CD40 in microglia, we treated both N9 and primary microglial cells with 25 μM of either apigenin or luteolin in the presence of IFN-γ and CD40 agonistic antibody. Following ELISA, we observed reductions in both secreted levels of TNF-α and IL-6 from flavonoid treated microglia as compared to positive control (Fig. 3). As shown in Figure 3, microglia co-treated with a stimulatory concentration of IFN-γ and CD40 agonistic antibody simulates activation, thereby markedly elevating secretion of the archetypical pro-inflammatory cytokines TNF-α and IL-6 and over negative control. However, in the presence of apigenin or luteolin TNF-α (Fig. 3A and 3B) and IL-6 (Fig. 3C and 3D) secretion is significantly reduced (p < 0.01). Therefore, when considering the above data, it is apparent that both apigenin and luteolin modulate microglial activation and may prevent microglial-mediated inflammation by suppression of CD40 signaling.

### Apigenin and luteolin inhibit IFN-γ-induced STAT1 phosphorylation in N9 and murine-derived primary microglial cells

**Figure 4:**
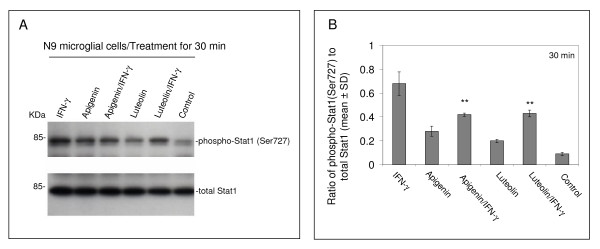
**Apigenin and luteolin oppose IFN-γ-induced phosphorylation of STAT1 in N9 microglial cells**. N9 murine microglial cells were seeded in 24-well tissue-culture plates (1 × 10^5^/well) and treated with IFN-γ (100 U/mL) in the presence or absence of apigenin and luteolin (25 μM) or treated with vehicle (1% DMSO; control) for 30 mins. Cell lysates were prepared from these cells and subjected to western immunoblotting using anti-phospho-STAT1 (Ser727) and anti-total STAT1 antibody as indicated. For B, data are represented as mean ratios of phospho-Stat1(Ser727) to total Stat1 (+/- SD) from densitometric analyses. Results are representative of three independent experiments. (**p < 0.01).

**Figure 5:**
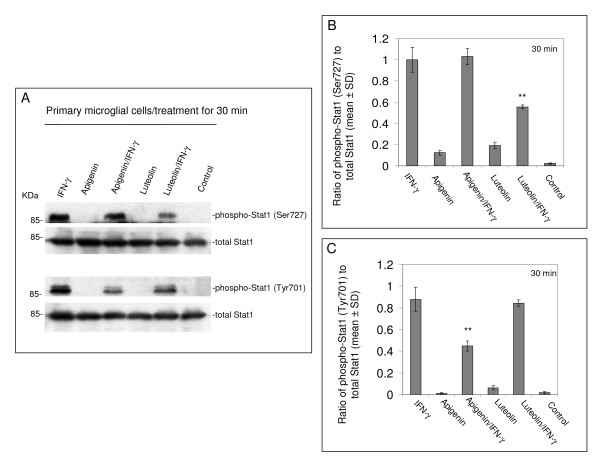
**Apigenin and luteolin oppose IFN-γ-induced phosphorylation of STAT1 in murine-derived primary microglial cells**. Murine-derived primary microglial cells were seeded in 24-well tissue-culture plates (1 × 10^5^/well) and treated with IFN-γ (100 U/mL) in the presence or absence of apigenin and luteolin (25 μM) or treated with vehicle (1% DMSO; control) for 30 mins. Cell lysates were prepared from these cells and subjected to western immunoblotting using anti-phospho-STAT1 (Ser727 or Tyr701) and anti-total STAT1 antibody as indicated. For B and C, data are represented as mean ratios of phospho-Stat1(Ser727 or Tyr701) to total Stat1 (+/- SD) from densitometric analyses. Results are representative of three independent experiments. (**p < 0.01).

To establish the mechanism whereby apigenin and luteolin decrease CD40 expression on the microglial cell surface, we investigated the upstream STAT1 signaling pathway. Both N9 and primary microglial cells were again treated with a 25 μM concentration of either apigenin or luteolin in the presence of a stimulatory amount of IFN-γ. Following western blot analysis of cell lysates, we found a decrease in STAT1 signaling in both flavonoid treatments as compared to positive control (Fig. 4 and 5). Densitometric analyis of western blot data from N9 cell lysates in Figure 4B indicate significant reductions in STAT1 phosphorylation at serine residue 727 (p < 0.01) with both apigenin and luteolin treatment. However, densitometric analysis of western blot data from primary microglial cells in Figure 5B and 5C reveals that apigenin significantly reduces STAT1 phosphorylation at tyrosine residue 701 (p < 0.01) and luteolin significantly reduces STAT1 phosphorylation at serine residue 727 (p < 0.01). Importantly, apigenin and luteolin treatment does not appear to affect holoprotein levels of STAT1 (Fig. 4A and 5A). Interestingly, we observed slight increases in STAT1 phosphorylation at serine 727 with flavonoid treatment alone, which may suggest these compounds have other unrelated, separate mechanisms. All in all, the above data suggest that apigenin and luteolin treatment regulates STAT1 at the level of phosphorylation and prevents the necessary signaling, in response to IFN-γ, required to induce CD40 expression.

## Discussion

Compounds that modulate microglial activity would be indispensable tools in neurodegenerative disease therapy. Here, we identify two flavonoids, apigenin and luteolin, that reduce CD40 expression on microglia and elucidate a mechanism whereby these compounds may prevent harmful neuroinflammation. The transcription factor STAT1 has been a potential therapeutic target in our previous neurodegeneration research. It is becoming evident that STAT1 and related signaling proteins may be regulated by flavonoids. Apigenin and luteolin appear to affect the phosphorylation status of tyrosine 701 and serine 727, which is required for STAT dimerization and maximal activation of transcription, respectively [53,54]. Even though we do observe slight increases in serine 727 STAT1 phosphorylation with flavonoid treatment alone (Fig. 4 and 5), it is likely that this phenomenon is related to these compounds' pro-apoptotic effects evidenced in tumor cell lines [55,56]. In a study by Elsisi and colleagues, apigenin was found to induce apoptosis in the BV-2 microglial cell line after 24 hours of incubation [57]. However, in our present study it is apparent that a 25 μM concentration of apigenin may be at worst cytostatic towards both the primary derived and the "transformed/immortalized" N9 microglia with treatment times under 24 hours (Fig. 2C and 2D). It has been reported that regulation of apoptosis via STAT1 is dependent upon serine 727 phosphorylation [58-60]. In contrast, we observe significant reductions in serine 727 phosphorylation with apigenin in N9 microglia and luteolin in both N9 and primary microglia in the presence of IFN-γ (Fig. 4 and 5). Therefore, it may be likely that the pro-apoptotic/anti-carcinogenic and immunomodulatory mechanisms of these flavonoids interface at the level of STAT1, a molecule qualified to function in this capacity. Still, distinct differences between these mechanisms may be dependent on the microenvironment and remain to be established.

While phosphorylation of tyrosine residue 701 on STAT1 is an event essential for STAT dimerizaton and translocation, it neither is a required precursor to serine 727 phosphorylation nor a mediator of apoptosis [58-60]. However, it appears to be as necessary as serine 727 phosphorylation for STAT1 signaling as apigenin effectively reduces CD40 expression in the presence of IFN-γ in primary microglia (Fig. 2B). Interestingly, we find that apigenin appears to regulate tyrosine 701 phosphorylation, but not serine 727 phosphorylation, of STAT1 in primary microglia, which suggests that it has a distinct target/mechanism separate from that of luteolin. Furthermore, these mechanistic differences underscore the differences inherent between primary derived and N9 microglia.

The ability of these flavonoids to regulate CD40-CD40L interaction and consequently pro-inflammatory mediators may be useful in the treatment of many inflammatory diseases. Nonetheless, inflammation in neurodegenerative disease represents a specific immunoprofile, involving discrete units made up of glial cells and neurons. Complete abolishment of CD40-CD40L interactions between microglia, astrocytes, and neurons may prove to be hazardous, as we have previously shown that ligation of CD40 protected neuronal cells from nerve growth factor-β (NGF-β) or serum withdrawal-induced injury and promoted differentiation [61]. Rather, modulation of immune reactions in CNS would appear to be the most appropriate course of action for treating neurodegenerative disease. While it would be quite difficult to achieve this modulation with conventional drugs, flavonoids appear to be uniquely fitted to this task as we observe their ability to affect multiple converging signaling pathways. For instance, apigenin and luteolin appear to regulate serine 727 phosphorylation of STAT1 at levels that are potentially conducive to apoptosis, all the while keeping a damper on levels in the presence of IFN-γ (Fig. 4 and 5). It is in this way that these compounds are essentially promoters of homeostasis.

It is quite clear from both the present study and previous research that apigenin and luteolin are potentially safe and effective immunomodulators, although their direct molecular targets have not been identified. What seems to be apparent is that these compounds prevent STAT1 signaling, which consequently may prevent the transcription of its target genes. Even as these compounds affect STAT1, it is more likely that upstream kinases or phosphatases are directly involved. Phosphatidylinositol 3-kinase (PI3K), its effector kinase Akt, JAK1, and JAK2 are all required for phosphorylation of both serine 727 and tyrosine 701 of STAT1 [62]. Accordingly, these kinases are all potential molecular targets of apigenin and luteolin. Previous reports also suggest that similar flavonoids mediate their effects through protein tyrosine phosphatases. Furthermore, CD45, a protein tyrosine phosphatase important for immune regulation and function, has been identified as an upstream regulator of STAT1 signaling via dephosphorylation of JAKs [63]. In light of these findings, it may be important to evaluate the effects of these flavonoids on other potential immunoregulatory signaling molecules to better ascertain both safety and efficacy.

While a major limitation in the use of these flavonoids as immunomodulators or anti-inflammatory agents is their low bioavailability, apigenin, in particular, may hold some promise as a suitable therapeutic agent. Recent pK studies suggest that apigenin exhibits a slow metabolism, absorption, and elimination phase (92 hours in rats) [64-66]. Moreover, in one human pK study, apigenin was also found to significantly reduce measures of oxidative stress in blood [67]. Like many aglycone forms of flavonoids, apigenin and luteolin potentially reach their molecular targets by passive diffusion through cell membranes. This means of cellular uptake may explain the onset (<30 mins) of STAT1 inactivation as we observed following apigenin and luteolin treatment (Fig. 3). Despite the possibility of sustainable therapeutic concentrations, future work with these compounds will certainly need to address blood-brain-barrier permeability.

## Conclusion

In summary, flavonoids apigenin and luteolin may prevent microglial-mediated inflammation by modulating the induction of CD40 in response IFN-γ. This decrease in expression modulates CD40-40L interactions, which are critical to microglial activation. Apigenin and luteolin's mechanism of action appears to be targeted at regulating STAT1 activation. These studies may lay the foundation for the development of neurodegenerative disease modifying therapies focused on preventing harmful inflammation while maintaining proper glia-neuron interactions.

## Abbreviations

Aβ: beta-amyloid; AD: Alzheimer's disease; ANOVA: analysis of variance; CD40L: CD40 ligand; CNS: central nervous system; FACS: fluorescence activated cell sorting; INF-γ: interferon gamma; IL-6: interleukin-6; JAK/STAT: janus kinase/signal transducer and activator of transcription; LDH: lactate dehydrogenase; NGF-β: nerve growth factor beta; ROS: reactive oxygen species; TNF-α: tumor necrosis factor alpha.

## Competing interests

PB and PRS are cofounders, and RDS and JT are scientific consultants for Natura Therapeutics, Inc. (Tampa, FL), a USF spin-out company. Natura Therapeutics manufactures a botanically derived dietary supplement that contains both apigenin and luteolin.

## Authors' contributions

KR assisted in the experimental design, analyzed the data, and completed the manuscript and figures. JE assisted in the experimental design, conducted LDH and Western blot experiments, and assisted in the completion of the manuscript. YB assisted in the experimental design and conducted FACS experiments. PB and PS assisted in the experimental design. RDS and JT conceived and directed the project. All authors read and approved the final manuscript.
